# Anaerobic ammonium oxidation coupled to iron(III) reduction catalyzed by a lithoautotrophic nitrate-reducing iron(II) oxidizing enrichment culture

**DOI:** 10.1093/ismejo/wrae149

**Published:** 2024-07-31

**Authors:** Hong-Bin Zhang, He-Fei Wang, Jia-Bo Liu, Zhen Bi, Ruo-Fei Jin, Tian Tian

**Affiliations:** Key Laboratory of Industrial Ecology and Environmental Engineering (Ministry of Education, China), School of Environmental Science and Technology, Dalian University of Technology, Dalian 116024, China; National Marine Environmental Monitoring Center, Laboratory of Island Ecological Environment Protection, Dalian 116023, China; Key Laboratory of Industrial Ecology and Environmental Engineering (Ministry of Education, China), School of Environmental Science and Technology, Dalian University of Technology, Dalian 116024, China; School of Environment Science and Engineering, Suzhou University of Science and Technology, Suzhou 215009, China; Key Laboratory of Industrial Ecology and Environmental Engineering (Ministry of Education, China), School of Environmental Science and Technology, Dalian University of Technology, Dalian 116024, China; Key Laboratory of Industrial Ecology and Environmental Engineering (Ministry of Education, China), School of Environmental Science and Technology, Dalian University of Technology, Dalian 116024, China

**Keywords:** NRFeOx, feammox, iron cycle, enrichment culture, nitrate-ammonium transition zones

## Abstract

The last two decades have seen nitrogen/iron-transforming bacteria at the forefront of new biogeochemical discoveries, such as anaerobic ammonium oxidation coupled to ferric iron reduction (feammox) and lithoautotrophic nitrate-reducing ferrous iron-oxidation (NRFeOx). These emerging findings continue to expand our knowledge of the nitrogen/iron cycle in nature and also highlight the need to re-understand the functional traits of the microorganisms involved. Here, as a proof-of-principle, we report compelling evidence for the capability of an NRFeOx enrichment culture to catalyze the feammox process. Our results demonstrate that the NRFeOx culture predominantly oxidizes NH_4_^+^ to nitrogen gas, by reducing both chelated nitrilotriacetic acid (NTA)-Fe(III) and poorly soluble Fe(III)-bearing minerals (γ-FeOOH) at pH 4.0 and 8.0, respectively. In the NRFeOx culture, Fe(II)-oxidizing bacteria of *Rhodanobacter* and Fe(III)-reducing bacteria of *unclassified_Acidobacteriota* coexisted. Their relative abundances were dynamically regulated by the supplemented iron sources. Metagenomic analysis revealed that the NRFeOx culture contained a complete set of denitrifying genes along with *hao* genes for ammonium oxidation. Additionally, numerous genes encoding extracellular electron transport-associated proteins or their homologs were identified, which facilitated the reduction of extracellular iron by this culture. More broadly, this work lightens the unexplored potential of specific microbial groups in driving nitrogen transformation through multiple pathways and highlights the essential role of microbial iron metabolism in the integral biogeochemical nitrogen cycle.

## Introduction

Terrestrial net primary productivity is often limited by the availability of nitrogen (N) fixed by microorganisms from atmospheric N_2_ [1]. Liberation of this N as NH_4_^+^ from decaying biomass triggers a complicated and organized suite of processes, driven by various microorganisms, that facilitate the return of N to the atmosphere. Typically, NH_4_^+^ is first oxidized to NO_3_^−^ by nitrifiers in the presence of oxygen, and the NO_3_^−^ is then reduced to N_2_ under anoxic conditions through denitrification [2]. Additionally, dissimilatory NO_3_^−^ reduction to NH_4_^+^ (DNRA) causes the retention of ammonium in the environment [3]. NH_4_^+^ can be converted to N_2_ through anaerobic ammonium oxidation coupled to nitrite reduction or iron(III) reduction, otherwise known as anammox or feammox [2, 4]. Feammox is a newly discovered biochemical pathway for nitrogen transformation in different ecosystems such as terrestrial [2, 5], marine [6], wetland [7, 8], and soil environments [4, 9, 10]. In this process, NH_4_^+^ is oxidized to end-products including NO_3_^−^, NO_2_^−^, and N_2_, using ferric iron [chelated Fe(III) or Fe(III) minerals] as electron acceptors [4]. Current research on microbially driven feammox is primarily focused on enrichment cultures [4–10]. Although the underlying mechanism of feammox catalyzed by enrichment cultures remains intricate, these groundbreaking studies emphasize the critical role of microbial interaction in the functioning of feammox.

In recent decades, the conversion of different nitrogen species through microbial iron-redox processes has garnered significant attention in various fields such as microbiology, biogeochemistry, and environmental science. Alongside the study of feammox, the role of lithoautotrophic nitrate-reducing ferrous iron-oxidation (NRFeOx) has also become a focal point for researchers [11, 12]. This microbially driven transformation of nitrogen and iron culture, known as enrichment culture KS, was initially identified from a freshwater sediment in 1996 [13]. Inspired by this study, researchers successively enriched similar NRFeOx cultures from diverse environments, such as wastewater treatment plant [14], pyrite-rich limestone aquifer [15], and other freshwater sediment [16]. These NRFeOx cultures use inorganic carbon sources (e.g. CO_2_ or bicarbonate) and can anaerobically reduce nitrate and nitrite using both dissolved ferrous ions and Fe(II)-bearing minerals (e.g. siderite and pyrite) as the sole electron donors [13–16].

The redox potentials of various Fe(II)–Fe(III) pairs lie intermediate to those of oxidized and reduced nitrogen species, creating the potential for a coupling between iron-related redox reactions and nitrogen species [17]. For instance, Fe_2_O_3_ hydrates and FeCO_3_ form a redox pair with a potential of ~+0.1 V at neutral pH, whereas all redox pairs involved in the nitrate reduction pathway are much more positive [13]. Fe(III)-bearing minerals, such as lepidocrocite [14], green rust [18], and ferrihydrite [15, 18], produced during the NRFeOx process, are bioavailable electron acceptors for the feammox process in the presence of ammonium. Conversely, the reduction of Fe(III) on these mineral surfaces releases highly reactive Fe^2+^, which forms a cation layer with a low redox potential at the mineral–water interface [19]. This Fe^2+^-rich layer can reduce electrostatic repulsion at the mineral–water interface, thereby facilitating nitrate and electron transfer [20]. This process contributes to nitrate reduction via denitrification or DNRA, with Fe^2+^ serving as the electron donor. Previous studies have reported that specific NRFeOx cultures enriched from wastewater treatment plants contain not only iron-oxidizing and denitrifying bacteria (e.g. species of *Gallionellaceae* and *Rhodanobacter*) but also microorganisms capable of ammonium oxidation and Fe(III) reduction, such as species of *Nitrosomonadaceae* and *Geothrix* [14, 21]. Therefore, it is reasonable to hypothesize that NRFeOx enrichment cultures could potentially facilitate the feammox process.

To test the hypothesis that NRFeOx enrichment cultures can anaerobically oxidize ammonium coupled to iron(III) reduction (feammox), we designed a comprehensive experiment to investigate this capability in an enriched NRFeOx culture. We analyzed the ammonium transformation pathway in this culture using a ^15^N stable isotope probing test. Additionally, we conducted metagenomic analyses of the NRFeOx culture under various cultivation conditions to compare the variations in functional genes involved in the transformation of nitrogen and iron species, thereby uncovering the mechanisms of interspecific interactions.

## Materials and Methods

### Enrichment of a nitrate-reducing ferrous iron-oxidation culture

The inoculum for enriching the NRFeOx culture was collected from a secondary sedimentation tank at the Xiajiahe Sewage Treatment Plant in Dalian, China. It was then cultivated in an up-flow anaerobic bioreactor in lab for culture enrichment (Fig. S1). The bioreactor was equipped with a stirred slurry (100 rpm), and KHCO_3_ was used as the inorganic carbon source. The growth medium consisted of (in mg L^−1^): KHCO_3_–500, KH_2_PO_4_-25, NaCl-50, MgSO_4_·7H_2_O-75, and CaCl_2_·H_2_O-20, and trace elements of (in μg L^−1^): H_3_BO_3_-14, NiCl_3_·6H_2_O-190, CuSO_4_·5H_2_O-250, MnCl_2_·4H_2_O-990, CoCl_2_·6H_2_O-240, NaMoO_4_·2H_2_O-220, ZnSO_4_·7H_2_O-430, and NaWO_4_·2H_2_O-50. NaNO_3_ (2 mM) and FeCl_2_ (10 mM) were supplemented to the medium as electron acceptor and donor, respectively. The initial pH of this medium was adjusted to 7.0 ± 0.1 using 0.1 M HCl, and the dissolved oxygen (DO) level was maintained below 0.5 mg L^−1^ by bubbling high-purity N_2_ for 20 min. The NRFeOx culture was continuously enriched at 30 ± 1°C for 180 days. Effluent parameters were monitored until they reached steady concentrations, at which point the molar ratio of reduced NO_3_^−^ to oxidized Fe(II) approached 0.2. The Fe(III) mineral products at the stable state were collected and identified by combining powder X-ray diffraction (XRD) and X-ray photoelectron spectroscopy (XPS).

### Feammox activity of nitrate-reducing ferrous iron-oxidation culture

The NRFeOx culture, harvested after 180 days of cultivation, was centrifuged at 8000 rpm for 10 min and subsequently rinsed three times with a 0.9% (w/v) NaCl saline buffer to remove any residual dissolved nitrogen and iron species. Approximately 1 g (wet-weight) of NRFeOx culture was transferred into each 100 ml serum vial containing 50 ml of the aforementioned growth medium, resulting in a volatile suspended solids of 1500 mg L^−1^. This growth medium contained 10 mM Fe(II) and 2 mM nitrate. The vials were sealed with butyl rubber septa and crimped with aluminum caps. After purging with N_2_/CO_2_ (90%/10%) for 20 min, they were incubated in a glove box flushed with high-purity N_2_ at 30 ± 1°C under dark conditions, to verify the activity of the NRFeOx culture. After an 8-day verification period, the feammox activity of the NRFeOx culture was assessed in the aforementioned growth medium (50 ml) under the following conditions: (i) Fe(III)-NTA (2.5 mM) and ammonium (1.5 mM); (ii) Fe(III)-NTA (2.5 mM) without ammonium; (iii) the absence of Fe(III)-NTA with ammonium (1.5 mM); and (iv) the NRFeOx culture containing produced γ-FeOOH minerals with ammonium (1.5 mM) under both acidic (pH = 4.0 ± 0.2) and basic (pH = 8.0 ± 0.2) conditions. Accordingly, samples were collected daily during these validation assays. The Fe(II)-oxidizing capability of these NRFeOx cultures after incubation under feammox conditions was further estimated.

All treatments were set up in triplicate, and abiotic groups including filter-sterilized medium (culture-free) were used as controls. The sterilized medium was obtained after being filtered through a 0.22 μm membrane filter. These activity assays lasted for 1 week, during which the concentrations of Fe(II), Fe(total), nitrate, nitrite, and ammonium in the growth media were monitored daily. After incubation, the collected solids were placed on petri dishes in the glove box to dry for 48 h, followed by vacuum drying at 70 ± 1°C for further XRD and XPS analyses.

### ^15^N-label-based rate measurements

Approximately 2 g (wet-weight) of NRFeOx culture, rinsed with a 0.9% (w/v) NaCl saline buffer, was transferred into 100 ml serum vials containing 50 ml of growth medium. After sealing, the headspace air in these vials was replaced with helium by bubbling with 99.999% He. Three different sets of experimental assays were conducted in triplicate: (i) ^15^N-labeled ammonium (^15^NH_4_Cl, 1.5 mM) and Fe(III)-NTA (2.5 mM); (ii) ^15^N-labeled ammonium (^15^NH_4_Cl, 1.5 mM) and γ-FeOOH (0.26 g); and (iii) unlabeled ammonium (NH_4_Cl, 1.5 mM). These vials were incubated in the N_2_-frushed glove box at 30 ± 1°C for 1 week. At the end of the incubation period, 20 ml of gas was collected from the headspace of each vial using a gastight syringe. This gas was then injected into vacuum-sealed 20 ml glass bottles (initially filled with helium before vacuuming) for the measurement of ^15^N-labeled N_2_. Details regarding the calculation of ^30^N_2_ and ^29^N_2_ production rates are described in Supplementary Information.

### DNA extraction and metagenome analysis

Genomic DNA was extracted from NRFeOx culture samples before and after feammox validation experiments using a E.Z.N.A™ Mag-Bind Soil DNA Kit (Omega, USA) following the manufacturer’s instructions. The extracted DNA samples were then prepared for shotgun metagenome sequencing using the MaxUp II DNA Library Prep Kit (Yeason, China). Before sequencing, the DNA concentration of each PCR product was determined using a Qubit 4.0 Green double-stranded DNA assay and it was quality-controlled using a bioanalyzer (Agilent 2100, USA). Depending on coverage needs, all libraries can be pooled for one run. The amplicons from each reaction mixture were pooled in equimolar ratios based on their concentration. The sequencing was performed on the Novaseq 6000 platform (Illumina) by Novogene (Beijing, China). A total of 94031978 (~9.01 Gb) and 116673426 (~11.27 GB) raw reads were obtained and qualified using Fastp v.0.36, with a minimum Phred quality threshold of 20 (Table S1). To identify possible contamination during the sequencing experiments, Bowtie2 v.2.1.0 was used to align the reads with human sequences. A total of 80567450 (~7.68 Gb) and 112562348 (~10.73 Gb) of clean data were assembled using SPAdes v.3.13, with contigs smaller than 500 bp filtered out. The resulting sequences were then clustered using CD-HIT v.2.60 with parameters set at 95% identity and 90% coverage to create a nonredundant gene catalog. This catalog was annotated with biological functions using DIAMOND v.0.8.20 by aligning the sequences with the KEGG database.

To group the metagenome-assembled genomes (MAGs), Maxbin 2 and metaBAT 2 algorithms in MetaWRAP v.1.3.0 were used with parameters set at -c 70, -x 10. The resulting bins were then annotated with genes and taxonomy using GTDB-Tk v. 2.3.0 and Prokka v.1.14.6 by comparing them with the National Center for Biotechnology Information (NCBI) database. To identify potential genes involved in extracellular electron transfer, the predicted protein sequences from the bins were searched against hidden Markov models (HMMs) using HMMER v.3.0. The raw sequencing data of the NRFeOx culture before and after feammox incubation have been successfully submitted to the NCBI BioProject database. The data are associated with the BioProject accession number PRJNA1041035. Additionally, the raw sequences data from the metagenome can be accessed in the Sequence Read Archive (SRA) under accession number SRR26965776 and SRR26974281 for the NRFeOx culture before and after feammox incubation, respectively.

### Chemical analysis methods

Volatile suspended solids (VSSs) were determined according to standard methods [22]. DO and pH were measured using a portable meter (WTW, multi-3630 IDS, Germany). Samples were collected from serum vials using sterile syringes and then centrifugated at 8000 rpm for 10 min at 4°C. The supernatant was collected to determine the concentrations of nitrate, nitrite, and ammonium using standard methods [22] with a UV-Visible spectrophotometer (MAPADA, P3, China). A modified ferrozine protocol, described by previously published studies [23, 24], was employed for the quantification of Fe(II) and Fe(III) in order to eliminate the influence of abiotic reaction between nitrite and Fe(II). Briefly, 100 μl samples were extracted from each vial using a sterile syringe and injected into 900 μl of 40 mM sulfamic acid in 1 M HCl. The mixture was then shaken for 1 h in the glove box under dark conditions. Subsequently, the samples were centrifuged at 8000 rpm to obtain the supernatant for the ferrozine assay.

The concentrations of N_2_ and ^15^N-labeled N_2_ (atom%) were determined using Isotope ratio mass spectrometry (Thermo Finnigan, Delta V Advantage, Germany) with an external connector, GasBench II. The mole fractions of ^29^N_2_ and ^30^N_2_, as well as the feammox rate, were calculated as described in the Supplementary Information. The vacuum-dried iron oxidation products collected before and after feammox process were identified using XRD and XPS. XRD analysis was carried out using an X-ray Powder diffractometer (Bruker, D8 advance, Germany), with Cu Ka radiation (l = 1.54718 Å), in the 2*q* range from 10° to 70°. XPS analysis was performed using a ThermoFisher X-ray photoelectron spectrometer (K-alpha plus, USA), to investigate the surface elemental states for Fe, C, and O.

## Results and Discussion

### Feammox driven by nitrate-reducing ferrous iron-oxidation culture

**Figure 1:**
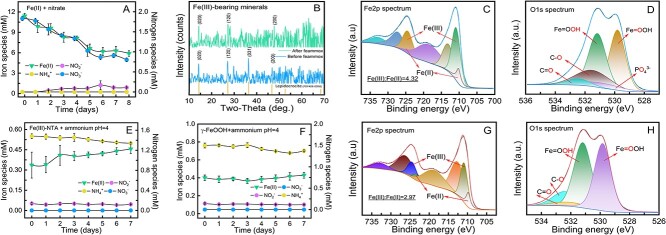
Feammox process catalyzed by NRFeOx culture. (A) NRFeOx. (B) XRD patterns of the Fe(III)-bearing minerals before/after feammox incubation. (C, D) XPS spectra of the Fe(III)-bearing minerals before feammox incubation. (E) Feammox verification (Fe(III)-NTA + NH_4_^+^) at pH = 4.0. (F) Feammox verification (Fe(III)-bearing minerals + NH_4_^+^) at pH = 4.0. (G, H) XPS spectra of the Fe(III)-bearing minerals after feammox incubation.

The NRFeOx enrichment culture reduced 1.0 mM of NO_3_^−^, concurrently oxidizing ~5.5 mM of Fe(II) (Fig. 1A). The observed ratio of reduced NO_3_^−^ to oxidized Fe(II) is ~0.18, which is close to the ideal stoichiometry of Fe(II) required for nitrate reduction and CO_2_ fixation [15, 25]. NO_2_^−^ and NH_4_^+^ are not detectable, indicating that this culture may have a high nitrite-reducing activity [15, 26] and the nitrate reduction is likely attributed to denitrification rather than DNRA [3]. XRD and XPS spectra revealed that the primary products of Fe(II) oxidation in the NRFeOx culture include the crystalline structure of layered lepidocrocite (γ-FeOOH) and amorphous Fe(III)-bearing minerals with poor crystallinity (Fig. 1B–D) [14, 27]. Furthermore, the Fe 2p XPS spectrum indicated that Fe(II) is closely associated with the Fe(III)-bearing minerals, likely through adsorption, coprecipitation, or other pathways (Fig. 1C). The Fe(III)-to-Fe(II) ratio was determined to be 4.32.

Although the feammox process can occur across a wide pH range, previous studies have indicated that the microorganisms (e.g. *Acidimicrobiaceae*) responsible for this process are commonly found in weakly acidic iron-rich environments [28, 29]. The NRFeOx process produces bioavailable Fe(III) species and creates an acidic environment [14, 15], potentially providing a “hotbed” for the occurrence of feammox process. Based on this consideration, we subsequently transferred the NRFeOx culture into a fresh growth medium (pH = 4.0) containing ammonium and chelated Fe(III) (Fe(III)-NTA) to verify its ability to perform the feammox process. The NRFeOx culture oxidized ~0.15 mM NH_4_^+^ without producing any detectable NO_2_^−^ and NO_3_^−^ (Fig. 1E). Concurrently, Fe(II) gradually accumulated to approximately 0.12 mM in the medium as ammonium was oxidized.

Either Fe(III) reduction or ammonium oxidation was observed in the abiotic controls lacking the NRFeOx culture (Fig. S2C, E, and F), but Fe(II) accumulation did occur in the group without ammonium (Fig. S2E). These results demonstrated that the NRFeOx culture possesses the capability to anaerobically oxidize ammonium and reduce chelated-Fe(III) in acidic environments. However, it remains unknown whether this culture could effectively reduce solid Fe(III)-bearing minerals, including those it produces, thereby completing a comprehensive iron cycle from Fe(II) to Fe(III) and vice versa. Subsequently, we utilized the *in situ* produced Fe(III)-bearing minerals (γ-FeOOH) as an electron acceptor to confirm this feasibility. Again, we observed a simultaneous Fe(II) generation and oxidation of ~0.12 mM of NH_4_^+^ (Fig. 1F). However, the accumulation of Fe(II) was considerably less than that observed in the Fe(III)-NTA addition group (Fig. 1E) and even lower than that observed in the control experiment without iron (Fig. S2D).

Compared to the Fe(III)-NTA group, these discrepancies were assumed to result from the *in situ* reduction of Fe(III) species on the surface of minerals immediately after being dissolved [30, 31]. The *in situ* reduction of Fe(III) can lead to the formation of an Fe^2+^-rich layer on the surface of iron minerals. This layer hinders the migration of Fe^2+^ into solution and reduces the redox potential at the mineral-water interface, thereby facilitating the further dissolution and reduction of Fe(III) [19, 20]. Consequently, although the bioavailability of solid iron minerals to microorganisms is lower compared to chelated iron, the ammonium oxidation rate in the γ-FeOOH system was comparable to that in the Fe(III)-NTA group, albeit with lower amounts of Fe(II) detected. After feammox cultivation, the *d*-spacing of the 031 plane of lepidocrocite became indistinguishable, indicating a decrease in crystallinity of the Fe(III)-bearing minerals (Fig. 1B). Moreover, the ratio of Fe(III) to Fe(II) in these minerals decreased to 2.97 (Fig. 1G), further providing strong evidence of Fe(III) reduction by the NRFeOx culture. Moreover, the NRFeOx culture retained its feammox capacity after being transferred to a new medium containing fresh Fe(III)-NTA and ammonium (Fig. S3).

The feammox process could also be influenced by pH, as it affects the products of this process by regulating the reactivity of iron minerals and their redox reactions [4, 8]. When the pH falls below 6.5, feammox microorganisms may consume more Fe(III) to produce NO_2_^−^ and NO_3_^−^ instead of N_2_. This shift is likely due to the increased thermodynamically favorability of the feammox reaction and the enhanced reactivity of Fe(III) minerals at lower pH levels [2]. This potential scenario raises questions about the feammox process we observed. Although we did not detect any NO_2_^−^ and NO_3_^−^ during the oxidation of NH_4_^+^, it is possible that after NH_4_^+^ is oxidized to NO_2_^−^ and NO_3_^−^, these species are immediately reduced by the *in situ* reduced Fe^2+^ (chemodenitrification) or by the NRFeOx culture using Fe^2+^ as electron donors. However, some evidence partially contradicts this possibility. In the Fe(III)-NTA group, the molar ratio of oxidized NH_4_^+^ to generated Fe(II) was ~1.25:1. Although this ratio is lower than the ideal stoichiometry, where 3 mol of Fe(II) are produced by oxidizing 1 mol of NH_4_^+^ [2, 8], it is considered acceptable. This discrepancy can be attributed to the electrons required for CO_2_ fixation by the NRFeOx culture and the potential adsorption of Fe(II) by minerals. To obtain more conclusive evidence and explore the NRFeOx culture’s ability to sustain feammox across a broader pH range, we further validated its feammox activity in a mildly alkaline environment (pH = 8.0).

As the pH increases, the hydrolysis of reduced ferrous ions to Fe(OH)^+^ and further precipitation of Fe(OH)_2_ occurs [32]. As a result, although the ammonium was obviously oxidized by the NRFeOx culture at pH = 8.0, the amount of Fe(II) generated was relatively limited (Fig. S4A). Similar results were also observed in the biotic group using Fe(III)-bearing minerals as electron acceptors (Fig. S4B). The reduction of Fe(III) was not observed in the abiotic groups without the NRFeOx culture, regardless of the presence of ammonium (Fig. S3E and S3F). After cultivation for 3 days, we observed unusual increases in NH_4_^+^ content in the NTA-amended groups, regardless of the presence of Fe(III) [the observed Fe(III) likely results from the chelation of iron species trapped in the NRFeOx culture by NTA, as shown in Fig. S4C]. In contrast, in the experimental groups with both Fe(NTA) and iron minerals, the NH_4_^+^ content consistently decreased (Fig. S4A and B). The ratios of oxidized NH_4_^+^ to reduced Fe(II) were determined to be 1.9 and 2.1, respectively. These findings demonstrate that the NRFeOx culture can facilitate the feammox process across a broader pH range. Further research is necessary to determine whether the unusual increase is due to microorganism lysis caused by the toxicity of NTA under alkaline conditions or other factors.

### ^30^N_2_ and ^29^N_2_ production from nitrate-reducing ferrous iron-oxidation culture

Certain members of NRFeOx cultures can fix CO_2_, providing potential carbon sources for those heterotrophic accompaniers (e.g. heterotrophic denitrifying bacteria) to sustain their survival [33–35]. This cooperative symbiotic system ensures the stability and functionality of the NRFeOx culture; however, it also raises the possibility of a dissimilatory iron reduction (DIR) process occurring within the culture. As a result, it remains debatable whether the observed reduction of Fe(III) to Fe(II) is due to feammox activity. Distinguishing between these two pathways within the NRFeOx system is challenging. To address this concern, we employed ^15^N-labeled ammonium-based isotope tracing techniques [4, 8, 9] to indirectly validate the iron reduction pathway by linking Fe(III) reduction to ammonium oxidation to N_2_.

**Figure 2:**
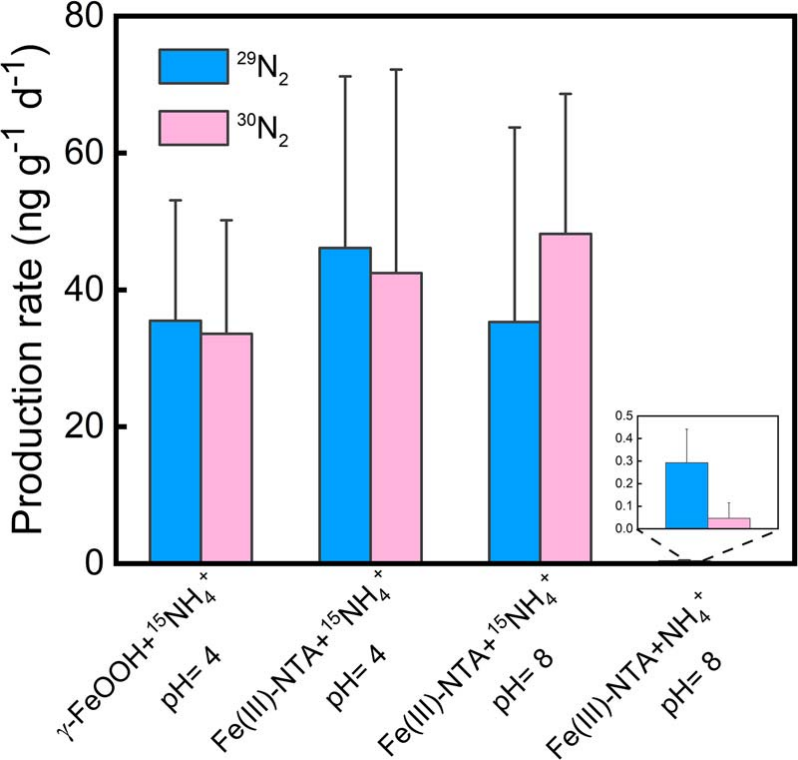
^29^N_2_ and ^30^N_2_ production rates in the NRFeOx culture incubated with different iron species using ^15^N-labeled ammonium-based isotope tracing technique. Error bars represent standard errors (*n* = 3).

Significant rates of ^30^N_2_ production were detected in all experimental treatments, including Fe(III)-NTA + ^15^NH_4_^+^ (pH = 4.0), Fe(III)-NTA + ^15^NH_4_^+^ (pH = 8.0), and Fe(III)-bearing minerals (γ-FeOOH) + ^15^NH_4_^+^ (Fig. 2). In comparison, the ^30^N production in the control group (without ^15^N-NH_4_^+^ added) was negligible. These results clearly demonstrated the occurrence of feammox process, because the production of ^30^N_2_ can only occur directly through feammox and/or indirectly through denitrification or anammox using the feammox-produced NO_2_^−^ and NO_3_^−^ under anoxic conditions [8, 9]. During the isotope-tracing incubations treated with ^15^NH_4_^+^, ^29^N_2_ also significantly accumulated in the systems. The similar production rates and variations of ^29^N_2_ and ^30^N_2_ indicate the combination of ^15^NH_4_^+^ with indigenous ^14^N species. These indigenous ^14^N species could include intracellular ^14^NH_4_^+^, which can be directly combined with added ^15^NH_4_^+^ to form ^29^N_2_ through feammox, or residual ^14^NO_3_^−^, which can combine with feammox-produced ^15^NO_3_^−^ to generate ^29^N_2_ through denitrification [8, 9]. Nevertheless, these pathways are all dependent on the initial reaction of anaerobic ^15^NH_4_^+^ oxidation. As a result, the primary cause of Fe(III) reduction catalyzed by the NRFeOx culture is through feammox rather than DIR.

### Recovery of nitrate-reducing ferrous iron-oxidation activity

As mentioned earlier, the NRFeOx culture can achieve anaerobic oxidation of NH_4_^+^ and denitrification of NO_3_^−^ through iron cycling. However, it is crucial for the NRFeOx culture to maintain its inherent metabolic capability even after being cultivated under feammox conditions. Maintaining Fe(II)-oxidizing ability over subculturing is one of the essential criteria for identifying true NRFeOx culture [11]. Previous research has shown that the enrichment culture KS retained the ability to oxidize Fe(II) during alternating autotrophic and heterotrophic cultivations, but this capability was lost after multiple transfers under exclusively heterotrophic conditions [18, 33].

**Figure 3:**
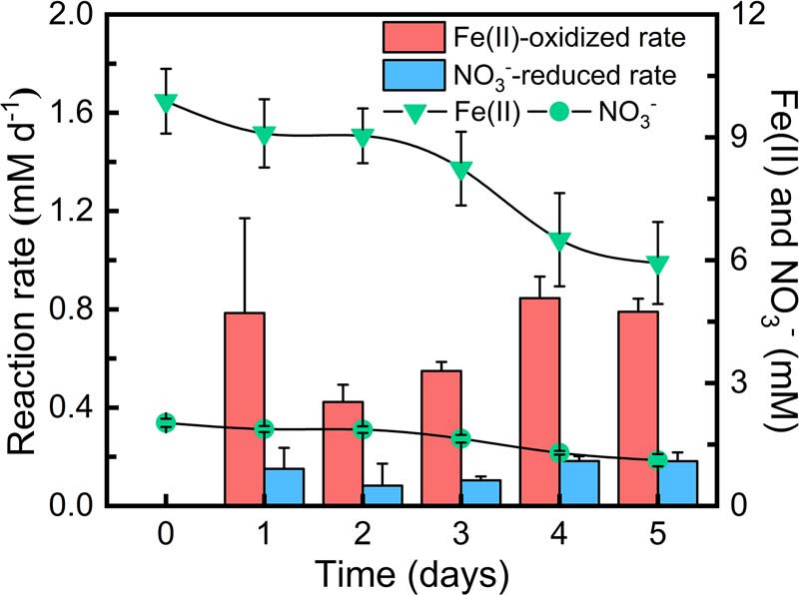
Recovery of inherent metabolic activity of the NRFeOx culture after feammox incubation.

To investigate whether the NRFeOx culture retained its metabolic capability of denitrifying with Fe(II) as the sole electron donor after being cultivated through feammox, we transferred the corresponding culture into fresh NRFeOx medium. We observed that compared to its initial incubation, this culture still exhibited a comparable NRFeOx activity, reducing ~0.18 mM of NO_3_^−^ and oxidizing 0.80 mM of Fe(II) per day (Fig. 3). Based on the findings discussed above, we conclude that the NRFeOx culture indeed possesses the capability to convert various nitrogen species through iron cycling. This suggests that in natural environments, especially in nitrate–ammonium transition zones [34], specific microbial communities with similar functions may have an unexpectedly significant role in the transformation of nitrogen species and the overall nitrogen cycle.

### Metabolic mechanisms based on metagenome analyses

In NRFeOx cultures, carbon fixation by iron-oxidizing bacteria plays a crucial role in supporting the survival of heterotrophic community members through metabolic cooperation [35, 36]. As a result, an NRFeOx culture typically consist of a high abundance of iron(II)-oxidizers that dominate the community, along with a relatively resilient and diverse flanking community. The results of metagenome taxonomy analysis reveal that bacteria make up the majority of both the NRFeOx culture before (~99.9%) and after feammox incubation (~99.8%). However, there was a decrease of Shannon index from 11.82 to 11.25, indicating the community diversity of the NRFeOx culture was decreased after feammox incubation (Table S2). This shift, at the phylum level, was primarily reflected in variations in the relative abundance of two phyla, *Pseudomonadota* and *Acidobacteriota* (Fig. S5A and B). Specifically, the relative abundance of *Pseudomonadota* decreased from 68.7% to 35.5%, whereas the relative abundance of *Aciobacteriota* increased to 43.9% from 8.7%.

The most dominant genus in the NRFeOx culture is *Rhodanobacter*, an iron-oxidizing bacterium [36], with a relative abundance of 16.3% (Fig. S5C). However, after feammox incubation, the abundance of *Rhodanobacter* significantly decreased to 2.6% (Fig. S5D). Despite previous findings that isolated *Rhodanobacter* sp. from culture KS could not independently oxidize Fe(II) [33], *Rhodanobacter* thrived as the dominant iron-oxidizing species in the NRFeOx culture. This success is likely due to metabolic cooperation with other community members, such as the carbon-fixing bacteria *Bradyrhizobium* and *Chloroflexota* [35, 36]. The most representative iron-oxidizing bacteria in previously identified NRFeOx enrichment cultures are *Gallinoellaceae* [13–15], which belong to the class of Betaproteobacteria. Although we did not explicitly identify this family at the genus level, the decrease in the abundance of *unclassified_Betaproteobacteria* may also be related to this shift. These findings further highlight the intricate interdependencies and synergistic relationships within the microbial community in the NRFeOx culture. The significant decrease in the relative abundances of *Rhodanobacter* and other identified autotrophic denitrifying iron oxidizers, such as *Hyphomicrobium* [37], suggests that the iron-dependent denitrification process was no longer the dominant mechanism in the NRFeOx culture.

The relative abundance of *unclassified_Acidobacteriota* increased from 7.5% to 35.2%, making it the most significantly increased genus. This genus includes a well-known iron(III) reducer, *Geothrix*, which has also been reported in a previous identified NRFeOx culture [14]. *Geothrix* is capable of reducing Fe(III) using various electron donors, including hydrogen, acetate, and lactate [38]. Furthermore, this genus has also been found to be involved in feammox process [4, 39]. Hence, it is plausible to suggest that the *unclassified_Acidobacteriota* identified in the metagenome may contribute to iron reduction in the NRFeOx culture during feammox incubation. However, we did not identify the typical feammox microbial species from *Acidimicrobiaceae* [29, 39], indicating the presence of other species responsible for ammonium oxidation in the NRFeOx culture.

**Figure 4:**
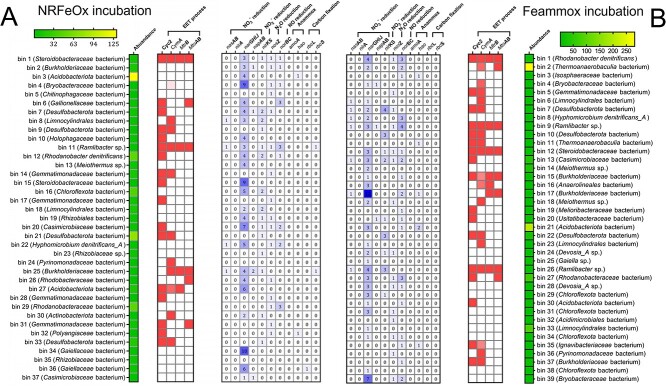
Heatmap of genes associated with denitrification, anammox, carbon fixation, and extracellular electron transport detected in the metagenomic bins of the NRFeOx culture before (A) and after feammox incubation (B). The color intensity in the genes associated with the EET process represents their respective E-values in HMM models, with darker colors indicating lower E-values.

At the genetic level, a total of 37 metagenome-assembled genomes (MAGs) were recovered from the NRFeOx culture (Fig. 4A), whereas 39 MAGs were obtained from this culture after feammox incubation (Fig. 4B). Genes encoding the denitrification process include *nasAB*, *narGHIJ*, *nirA*, *napAB*, *nirK/S*, *nosZ*, and *norBC* (Tables S3 and S4). In the NRFeOx culture, only two MAGs, bin 11 (*Ramlibacter* sp.) and bin 20 (*Casimicrobiaceae* bacterium), were found to possess a complete set of denitrifying genes. Other denitrifying bacteria in the culture were found missing some of the denitrification genes (Fig. 4A). For instance, bin 12 (*Rhodanobacter denitrificans*) lacks the *nosZ* gene, which is responsible for the reduction of N_2_O. Bacteria possessing NO-reducing genes are known to play a crucial role in the detoxification of NO, which is essential for the survival of other members within the NRFeOx culture [36]. After feammox incubation, the number and relative abundance of denitrifying genes in the culture remained high (Fig. 4B). These results further demonstrated the preservation of its inherent denitrification activity of the culture after feammox incubation (Fig. 3).

In terms of NH_4_^+^ oxidation, we detected the presence of *hao* genes, which encodes hydroxylamine oxidoreductase in the MAGs of the NRFeOx culture. However, we did not find the *amoA* genes for ammonia monooxygenase or *hzs* genes for hydrazine synthase involved in the anammox process. We speculate that the NH_4_^+^ oxidation pathway of the NRFeOx culture may be similar to those observed in anammox bacteria [40] or electroactive nitrifying microorganisms [41], which oxidized NH_4_^+^ anaerobically to hydroxylamine, coupled with extracellular electron transfer.

Iron-reducing bacteria utilize extracellular electron transport mechanisms to reduce Fe(III) associated with poorly soluble iron-bearing minerals [42, 43]. The process of extracellular electron transport relies on the involvement of transmembrane electron transporters, such as CymA and MtrB in Fe(III)-reducers [43, 44] and Cyc2 and MtoAB in Fe(II)-oxidizers [44, 45]. In the NRFeOx culture, we identified a total of 23 MAGs and 25 MAGs containing genes encoding these extracellular electron transport associated proteins or homologs before and after feammox incubation, respectively. These findings highlight the presence of a diverse range of bacteria in this culture capable of facilitating extracellular electron transport. We detected Cyc2- and MtoAB-like proteins with iron-oxidizing functions in *Rhodanobacter* and *Gallionellaceae* [36]. CymA- and MtrB-like proteins, known for their role in iron reduction, were also identified in *Rhodanobacter* after feammox incubation. This finding suggests that their function in NRFeOx enrichment cultures may have been previously overlooked [36, 44]. It is possible that these bacteria have the potential to simultaneously drive Fe(II) oxidation and Fe(III) reduction. Similar bacteria include *Ramlibacter*, which also possess these extracellular electron transport–associated proteins and has been identified as a chemoautotrophic manganese-oxidizing bacteria [46].

**Figure 5:**
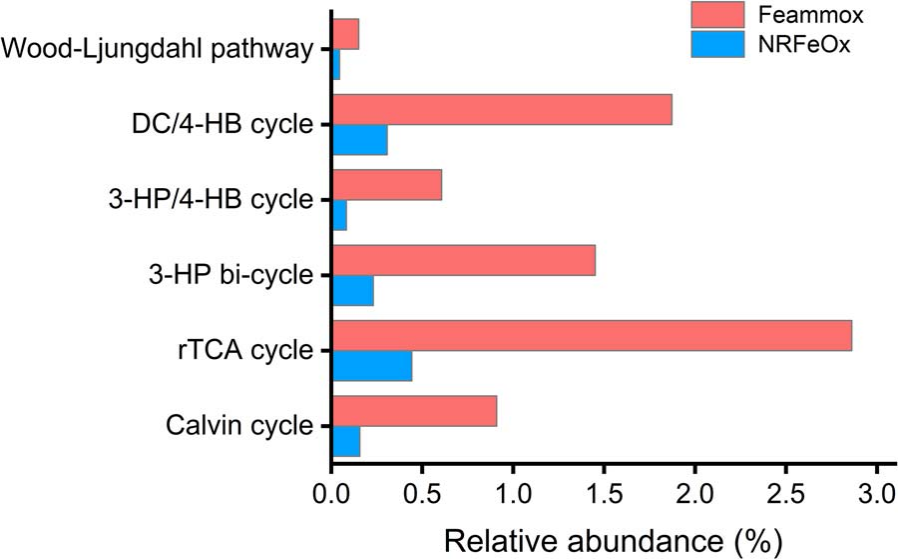
Variations of abundance of genes associated with carbon fixation pathways detected in the metagenome of the NRFeOx culture before and after feammox incubation.

Carbon fixation supports the survival and growth of the NRFeOx culture [35]. Initially, we detected a few *rbcL* and *rbcS* genes, which are responsible for CO_2_ fixation, in three MAGs: bin 11 (*Ramlibacter* sp.), bin 25 (*Burkholderiaceae* bacterium), and bin 36 (*Gaiellaceae* bacterium). These genes were not detected anymore after feammox incubation. However, in the analysis of microbial diversity (Fig. S5), we observed an increase in the relative abundance of *unclassified_Burkholderiaceae* to 3.2%, as well as an increase in other bacteria capable of fixing CO_2_, such as *unclassified_Chloroflexota*, which increased from 4.1% to 5.4% after feammox incubation. Therefore, we focused our analysis on the main CO_2_ fixation pathways present in the NRFeOx culture, including the Calvin cycle, reverse tricarboxylic acid (rTCA) cycle, 3-hydroxypropionate (3-HP) bi-cycle, 3-hydroxypropionate/4-hydroxybutyrate (3HP/4HB) cycle, dicarboxylate-4-hydroxybutyrate (DC/4HB) cycle, and Wood–Ljungdahl pathway [47, 48]. These pathways are crucial for converting CO_2_ or bicarbonate into organic carbon and are all included in the NRFeOx culture to support the growth and survival of the microbial consortium (Fig. 5). After feammox incubation, the abundance of genes involved in carbon fixation pathways in the culture was significantly increased. This increase suggests that the NRFeOx culture exhibits an enhanced carbon fixation activity during feammox incubation. Compared to the complete NRFeOx process (−96.23 kJ mol^−1^) [26], feammox to N_2_ yields much more energy (−245 kJ mol^−1^) [2], which could generate sufficient adenosine triphosphate to meet the energy demands of both the DC/4-HB cycle and 3-HP bi-cycle for CO_2_ fixation [49, 50]. Given that the NRFeOx culture is incubated under anoxic and dark conditions, it is likely that the rTCA cycle serves as the primary pathway for CO_2_ fixation. The rTCA cycle relies on the electron transport chain, and a substantial number of genes related to electron transport chain components have been identified within the NRFeOx culture. This genetic richness may facilitate the efficiency of the rTCA cycle in this culture. The fixed carbon can either be used for biomass assimilation, promoting growth, or be consumed for dissimilatory reduction of Fe(III) species, further facilitating feammox [50].

## Conclusions

This study demonstrated an NRFeOx enrichment culture drives the feammox metabolic pathway and provided a mechanistic understanding of how this microbial consortium achieves this process. The culture maintained its inherent capabilities for nitrate reduction and iron(II) oxidation. This property suggests that NRFeOx cultures can play essential roles in the transformation of nitrogen species in natural nitrate–ammonium transition zones where iron species are present. Looking ahead, NRFeOx cultures may serve as a bridge between natural and artificial ecosystems, closely linking these previously separate systems and offering valuable insights for microbial ecologists and applied microbiologists.

## Supplementary Material

Supplementary_Information_wrae149

## Data Availability

The raw sequencing data of the NRFeOx culture before and after feammox incubation have been successfully submitted to the NCBI BioProject database (PRJNA1041035). Additionally, the raw sequences data from the metagenome can be accessed in the Sequence Read Archive (SRA) under accession number SRR26965776 and SRR26974281 for the NRFeOx culture before and after feammox incubation, respectively.
