# Supplementary material for: Anaerobic ammonium oxidation coupled to iron(III) reduction catalyzed by a lithoautotrophic nitrate-reducing iron(II) oxidizing enrichment culture
Source: ISME J. 2024 Jul 31;18(1):wrae149. doi: 10.1093/ismejo/wrae149 (PMC11366258; doi:10.1093/ismejo/wrae149)
Supplement: Supplementary_Information_wrae149 [file supplementary_information_wrae149.docx]

***Supplementary Information for***

**Anerobic ammonium oxidation coupled to iron(III) reduction catalyzed by a lithoautotrophic nitrate-reducing iron(II) oxidizing enrichment culture**

Hong-Bin Zhang^1^, He-Fei Wang^2^, Jia-Bo Liu^1^, Zhen Bi^3^, Ruo-Fei Jin^1^, Tian Tian^1,*^

^1^Key Laboratory of Industrial Ecology and Environmental Engineering (Ministry of Education, China), School of Environmental Science and Technology, Dalian University of Technology, Dalian, 116024, China. ^2^National Marine Environmental Monitoring Center, Dalian 116023, P.R. China. ^3^School of Environment Science and Engineering, Suzhou University of Science and Technology, Suzhou 215009, China

^*^ Corresponding author: [skyetian@dlut.edu.cn](mailto:skyetian@dlut.edu.cn\)

**This Supplementary Information includes:** Supplementary text, Fig. S1 to S5, Table S1 to S4, and References

Supplementary Text

**Calculation of ^29^N_2_ and ^30^N_2_ production rate**

Mole fractions of ^29^N_2_ and ^30^N_2_ were calculated according to 29R and 30R obtained from IRMS as the following equations:

$$\begin{aligned} \text{f}_{\text{29}}\text{=}\frac{{}_{\text{ }}^{\text{29}}{\text{N}_{\text{2}}}}{{}_{\text{ }}^{\text{28}}{\text{N}_{\text{2}}\text{+}{}_{\text{ }}^{\text{29}}{\text{N}_{\text{2}}}\text{+}{}_{\text{ }}^{\text{30}}{\text{N}_{\text{2}}}}}\text{=}\frac{\frac{{}_{\text{ }}^{\text{29}}{\text{N}_{\text{2}}}}{{}_{\text{ }}^{\text{28}}{\text{N}_{\text{2}}}}}{\text{1+}\frac{{}_{\text{ }}^{\text{29}}{\text{N}_{\text{2}}}}{{}_{\text{ }}^{\text{28}}{\text{N}_{\text{2}}}}\text{+}\frac{{}_{\text{ }}^{\text{30}}{\text{N}_{\text{2}}}}{{}_{\text{ }}^{\text{28}}{\text{N}_{\text{2}}}}}\text{=}\frac{\text{29R}}{\text{1+29R+30R}}\#\left( \text{1} \right) \end{aligned}$$

$$\begin{aligned} \text{f}_{\text{30}}\text{=}\frac{{}_{\text{ }}^{\text{30}}{\text{N}_{\text{2}}}}{{}_{\text{ }}^{\text{28}}{\text{N}_{\text{2}}\text{+}{}_{\text{ }}^{\text{29}}{\text{N}_{\text{2}}}\text{+}{}_{\text{ }}^{\text{30}}{\text{N}_{\text{2}}}}}\text{=}\frac{\frac{{}_{\text{ }}^{\text{30}}{\text{N}_{\text{2}}}}{{}_{\text{ }}^{\text{28}}{\text{N}_{\text{2}}}}}{\text{1+}\frac{{}_{\text{ }}^{\text{29}}{\text{N}_{\text{2}}}}{{}_{\text{ }}^{\text{28}}{\text{N}_{\text{2}}}}\text{+}\frac{{}_{\text{ }}^{\text{30}}{\text{N}_{\text{2}}}}{{}_{\text{ }}^{\text{28}}{\text{N}_{\text{2}}}}}\text{=}\frac{\text{30R}}{\text{1+29R+30R}}\#\left( \text{2} \right) \end{aligned}$$

The total amount of N_2_ in serum vials filled with Helium was seemed as generated N_2_. The quality of N_2_ was calculated as equation 3 [1].

$$\begin{aligned} \text{M=}\text{ρ}_{\text{N}_{\text{2}}}\text{×V}\#\left( \text{3} \right) \end{aligned}$$

where $\text{M}$ is the quality of N_2_ in serum vials, $\text{ρ}_{\text{N}_{\text{2}}}$is the density of N_2_ at one atmosphere (usually considered to be 1.25 mg L^-1^), and $\text{V}$ is the volume of N_2_ transferred according to signal strength of the peak. The ^29^N_2_ and ^30^N_2_ production rates (P_29_ and P_30_) were calculated as equations 4 and 5 [2].

$$\begin{aligned} \text{P}_{\text{29}}\text{=}\frac{\text{M×}\left( \text{f}_{\text{29,t}}\text{-}\text{f}_{\text{29,0}} \right)}{\text{t×}\text{M}_{\text{sludge}}}\#\left( \text{4} \right) \end{aligned}$$

$$\begin{aligned} \text{P}_{\text{30}}\text{=}\frac{\text{M×}\left( \text{f}_{\text{30,t}}\text{-}\text{f}_{\text{30,0}} \right)}{\text{t×}\text{M}_{\text{sludge}}}\#\left( \text{5} \right) \end{aligned}$$

where $\text{f}_{\text{29,t}}$ and $\text{f}_{\text{30,t}}$are the mole fractions of ^29^N_2_ and ^30^N_2_ at t days, $\text{f}_{\text{29,0}}$ and $\text{f}_{\text{30,0}}$ are the mole fractions of ^29^N_2_ and ^30^N_2_ at 0 day, t is set as 7 in this study, and $\text{M}_{\text{sludge}}$ is the weight of sludge.


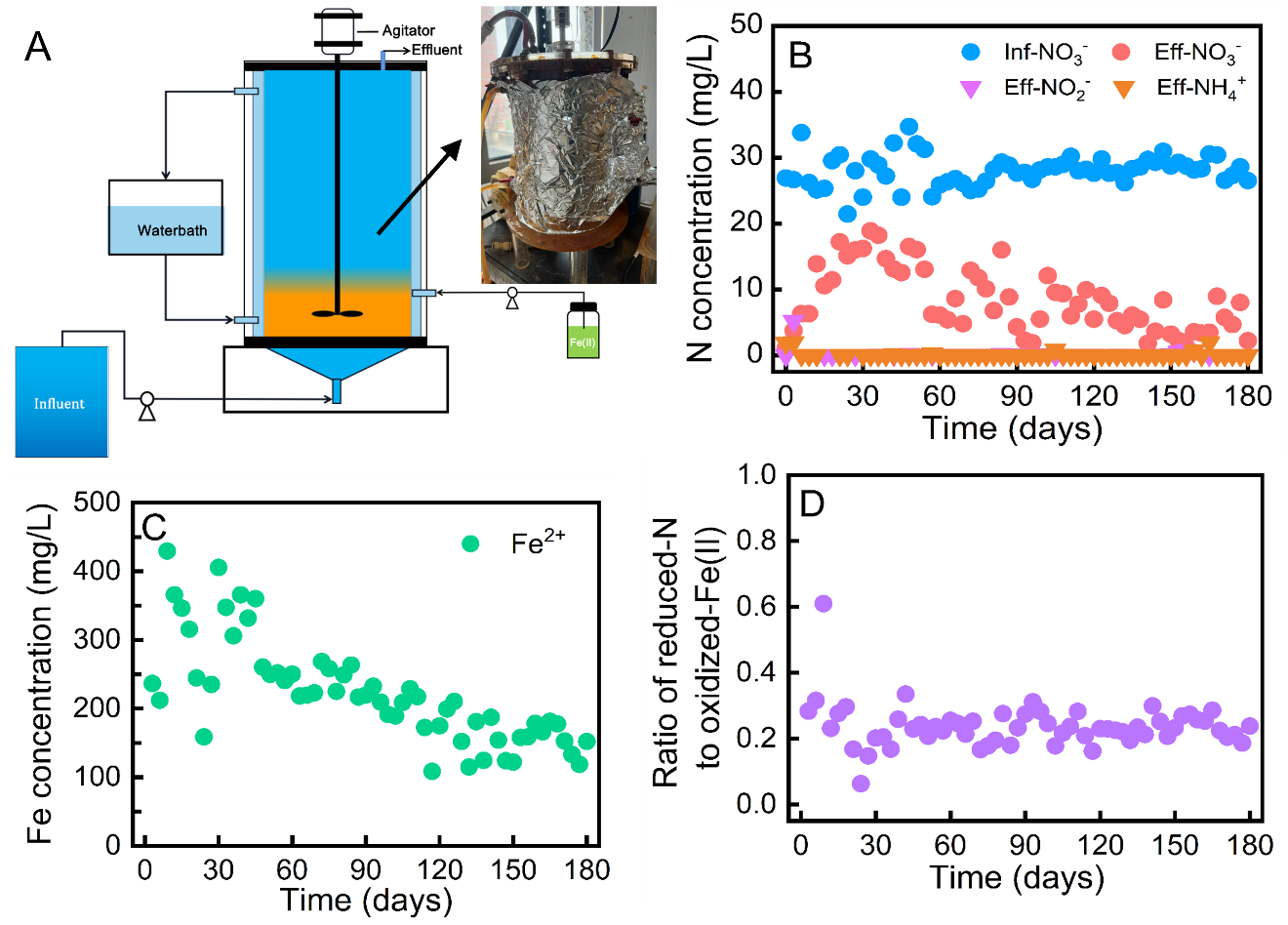


Fig. S1 Continuous enrichment of NRFeOx culture. A Schematic diagram and picture of the NRFeOx bioreactor. B Influent and effluent concentrations of nitrogen species. C Concentration of Fe^2+^ in the effluent. D Ratio of reduced-nitrate to oxidized-Fe(II).


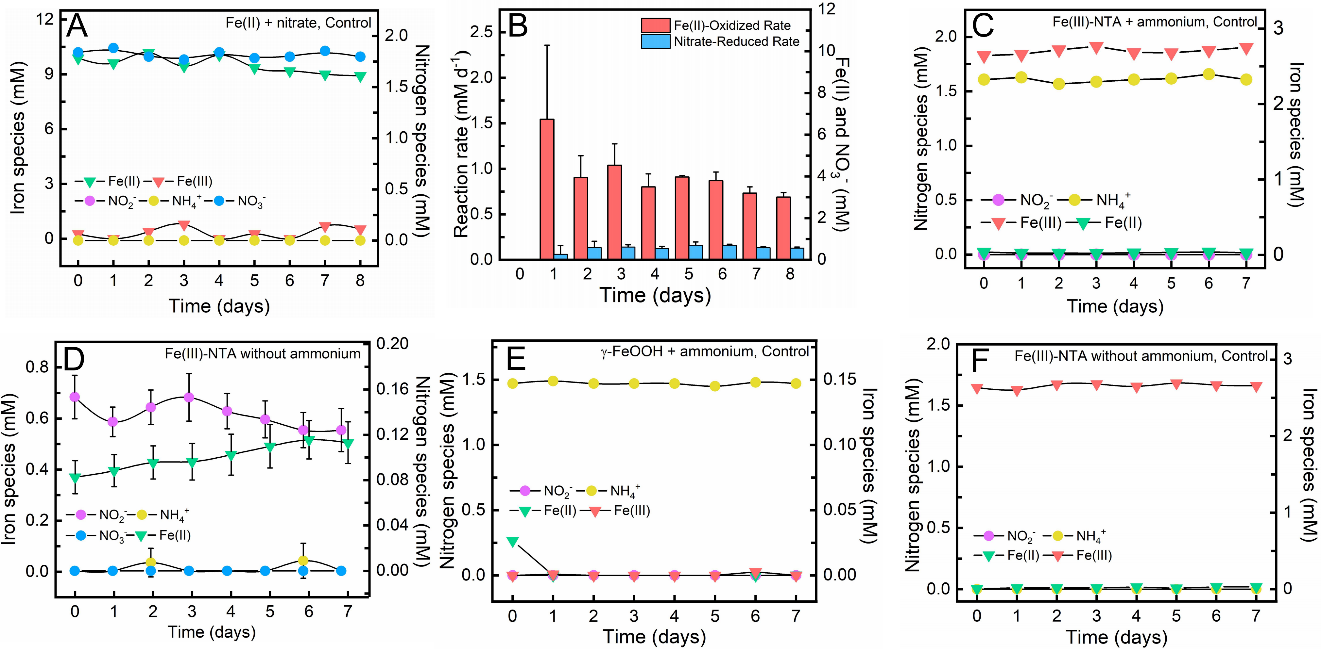


Fig. S2 A series of verification tests for feammox activity of NRFeOx culture. A Abiotic control of NRFeOx activity (without culture). B Reaction rate of NRFeOx. C Abiotic control of feammox activity (without culture). D Feammox verification (without NH_4_^+^). E Abiotic control of feammox (without Fe(III) and culture). F Abiotic control of feammox (without NH_4_^+^ and culture). Verification tests were conducted at pH 4.0.


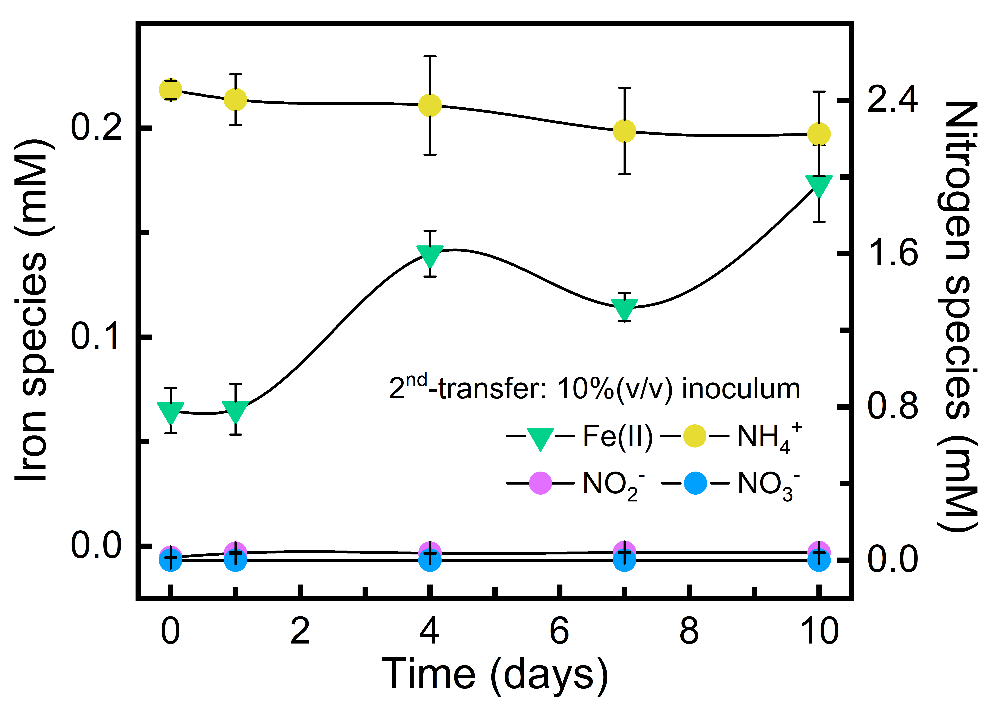


**Fig. S3. Feammox activity of NRFeOx culture in a 2^nd^-transfer incubation.**


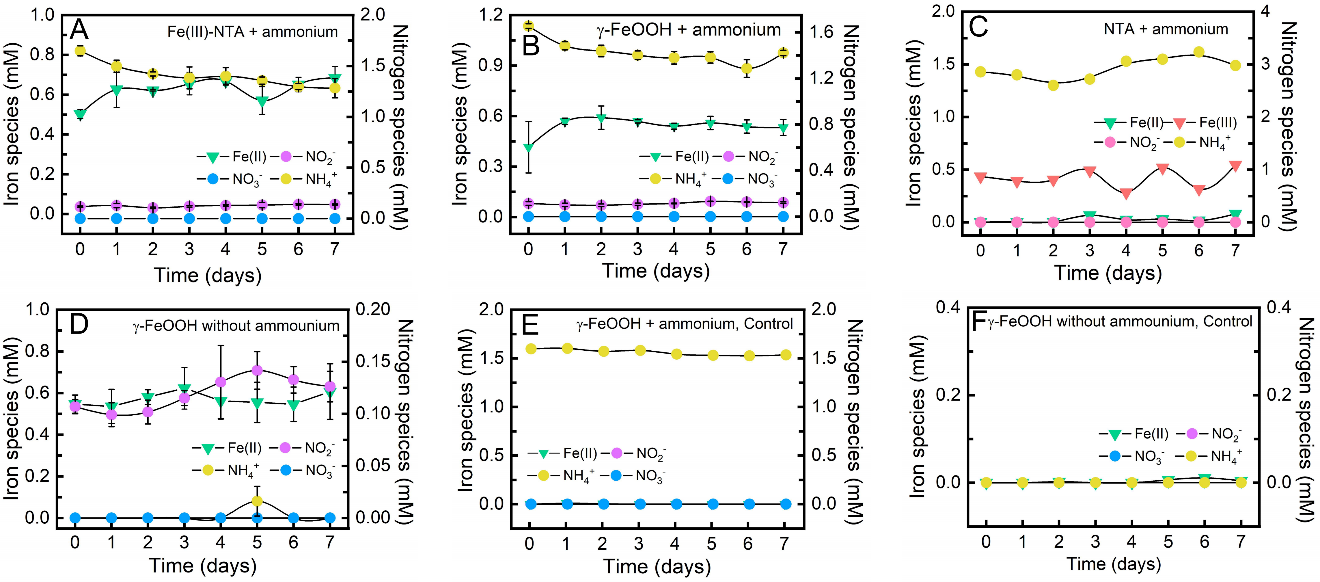


Fig. S4 NRFeOx culture catalyzes feammox process at pH 8.0. A Fe(III)-NTA + NH_4_^+^. B NH_4_^+^ without Fe(III)-NTA. C NTA + NH_4_^+^. D Fe(III)-bearing minerals without NH_4_^+^. E Fe(III)-bearing minerals + NH_4_^+^ without culture. F Fe(III)-bearing minerals without NH_4_^+^ and culture.


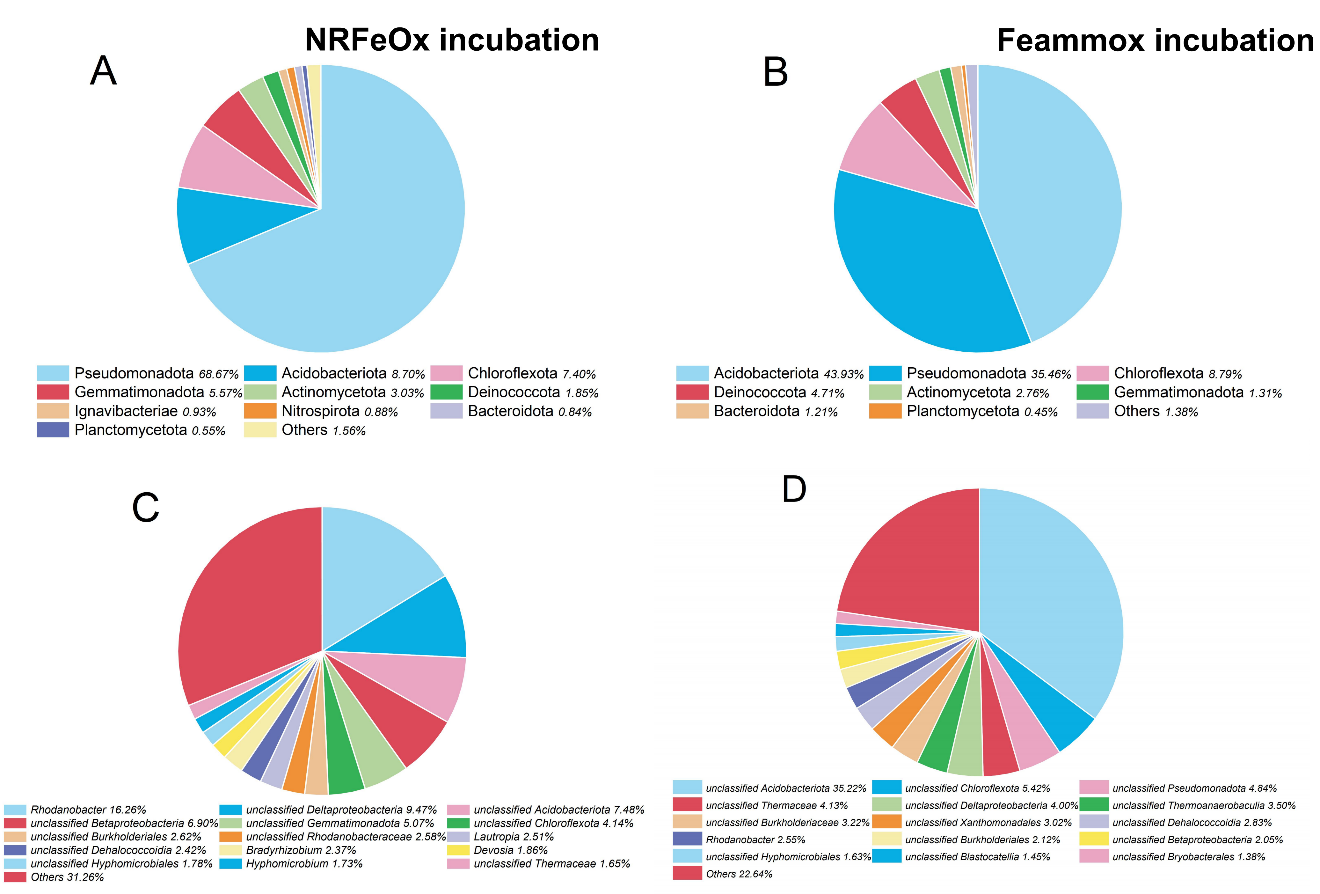


Fig. S5 Taxonomic classification of bacterial 16S rRNA gene reads of the NRFeOx culture at phylum level (A,B) and genus level (C,D) .

Table S1. Metagenome sequencing and assembly evaluate statistics.

| **Sequencing** | **NRFeOx** | **Feammox** |
| --- | --- | --- |
| Total read counts (#) | 94,031,978 | 116,673,426 |
| Average read length (bp) | 151 | 150 |
| Total raw sequence (Gb) | 9.01 | 11.27 |
| **Assembly** |  |  |
| Total No. of contigs | 309,028 | 299,319 |
| N50 (bp) | 2,138 | 2,825 |
| N70 (bp) | 1,038 | 1,213 |
| N90 (bp) | 631 | 643 |
| Max length (bp) | 266,183 | 640,274 |
| Average (bp) | 1,516 | 1,719 |
| Total length (bp) | 468,550,399 | 514,573,905 |
| GC content (%) | 64.07 | 63.83 |

Note: NRFeOx and Feammox represent the cultures before and after feammox incubation.

**Table S2**. **Alpha diversity of the microbial communities in NRFeOx cultures.**

| **Sample** | OTU | ACE | Chao1 | Shannon | Simpson | Coverage |
| --- | --- | --- | --- | --- | --- | --- |
| **NRFeOx** | 597,958 | 614778.80 | 609590.60 | 11.83 | 0.999974 | 0.998662 |
| **Feammox** | 648,378 | 655754.47 | 653476.52 | 11.25 | 0.999947 | 0.999788 |

Note: NRFeOx and Feammox represent the cultures before and after feammox incubation.

**Table S3.** **Read counts of denitrifying genes assigned into the top 15 genera of the initial NRFeOx culture.**

| Genus  in NRFeOx culture | NO_3_^-^ reduction | | | | | | | | NO_2_^-^ reduction | | NO  reduction | | N_2_O reduction |
| --- | --- | --- | --- | --- | --- | --- | --- | --- | --- | --- | --- | --- | --- |
|  | *nasA* | *nasB* | *narG* | *narH* | *narI* | *narJ* | *napA* | *napB* | *nirK* | *nirS* | *norB* | *norC* | *nosZ* |
| *Rhodanobacter* | 0 | 0 | 2453 | 1612 | 401 | 291 | 0 | 0 | 1952 | 0 | 3540 | 0 | 2088 |
| *unclassified Deltaproteobacteria* | 0 | 0 | 0 | 0 | 0 | 0 | 0 | 0 | 0 | 0 | 0 | 0 | 0 |
| *unclassified*  *Acidobacteriota* | 0 | 0 | 0 | 0 | 0 | 0 | 0 | 0 | 0 | 0 | 0 | 0 | 0 |
| *unclassified Betaproteobacteria* | 0 | 0 | 159 | 153 | 0 | 0 | 10 | 0 | 20 | 68 | 0 | 0 | 0 |
| *unclassified*  *Gemmatimonadota* | 0 | 0 | 0 | 45 | 0 | 0 | 0 | 0 | 0 | 0 | 0 | 0 | 0 |
| *unclassified*  *Chloroflexota* | 16 | 0 | 7 | 0 | 0 | 0 | 0 | 0 | 0 | 0 | 0 | 0 | 0 |
| *unclassified*  *Burkholderiales* | 0 | 0 | 704 | 329 | 3 | 0 | 7 | 0 | 0 | 6 | 0 | 0 | 0 |
| *unclassified*  *Rhodanobacteraceae* | 0 | 0 | 0 | 0 | 0 | 0 | 0 | 0 | 0 | 0 | 0 | 0 | 0 |
| *Lautropia* | 0 | 0 | 0 | 123 | 134 | 0 | 0 | 0 | 0 | 268 | 0 | 0 | 399 |
| *unclassified*  *Dehalococcoidia* | 0 | 0 | 0 | 0 | 0 | 0 | 0 | 0 | 0 | 0 | 0 | 0 | 0 |
| *Bradyrhizobium* | 12 | 0 | 7 | 187 | 11 | 0 | 90 | 10 | 41 | 0 | 5 | 0 | 159 |
| *Devosia* | 0 | 0 | 169 | 272 | 0 | 0 | 0 | 0 | 0 | 0 | 0 | 0 | 0 |
| *unclassified*  *Hyphomicrobiales* | 0 | 0 | 123 | 302 | 0 | 0 | 0 | 0 | 0 | 0 | 0 | 0 | 10 |
| *Hyphomicrobium* | 135 | 0 | 70 | 106 | 55 | 22 | 0 | 0 | 69 | 5 | 67 | 17 | 110 |
| *unclassified*  *Thermaceae* | 0 | 0 | 0 | 0 | 0 | 0 | 0 | 0 | 0 | 0 | 0 | 0 | 0 |

**Table S4.** **Read counts of denitrifying genes assigned into the top 15 genera of NRFeOx culture after feammox incubation.**

| Genus  in NRFeOx culture | NO_3_^-^ reduction | | | | | | | | NO_2_^-^ reduction | | NO  reduction | | N_2_O reduction |
| --- | --- | --- | --- | --- | --- | --- | --- | --- | --- | --- | --- | --- | --- |
|  | *nasA* | *nasB* | *narG* | *narH* | *narI* | *narJ* | *napA* | *napB* | *nirK* | *nirS* | *norB* | *norC* | *nosZ* |
| *unclassified*  *Acidobacteriota* | 0 | 0 | 0 | 0 | 0 | 0 | 0 | 0 | 0 | 0 | 35 | 0 | 0 |
| *unclassified*  *Chloroflexota* | 42 | 0 | 30 | 14 | 0 | 0 | 0 | 0 | 0 | 0 | 0 | 0 | 36 |
| *unclassified*  *Pseudomonadota* | 0 | 0 | 421 | 50 | 0 | 0 | 0 | 0 | 31 | 0 | 0 | 0 | 0 |
| *unclassified*  *Thermaceae* | 0 | 0 | 2479 | 1131 | 0 | 0 | 0 | 0 | 0 | 0 | 0 | 0 | 0 |
| *unclassified*  *Deltaproteobacteria* | 0 | 0 | 157 | 54 | 0 | 0 | 0 | 0 | 0 | 0 | 0 | 0 | 0 |
| *unclassified*  *Thermoanaerobaculia* | 0 | 0 | 0 | 0 | 0 | 0 | 0 | 0 | 0 | 0 | 0 | 0 | 0 |
| *unclassified*  *Burkholderiaceae* | 0 | 0 | 1291 | 599 | 224 | 0 | 0 | 0 | 0 | 428 | 0 | 0 | 500 |
| *unclassified*  *Xanthomonadales* | 0 | 0 | 71 | 2101 | 13 | 0 | 0 | 0 | 30 | 0 | 0 | 0 | 0 |
| *unclassified*  *Dehalococcoidia* | 23 | 0 | 0 | 0 | 0 | 0 | 0 | 0 | 0 | 0 | 0 | 0 | 0 |
| *Rhodanobacter* | 0 | 0 | 1332 | 268 | 218 | 186 | 0 | 0 | 1025 |  | 2234 | 0 | 3135 |
| *unclassified*  *Burkholderiales* | 0 | 0 | 1023 | 245 | 81 | 5 | 0 | 0 | 0 | 0 | 18 | 0 | 62 |
| *unclassified Betaproteobacteria* | 0 | 0 | 604 | 0 | 0 | 0 | 7 | 0 | 377 | 0 | 0 | 0 | 0 |
| *unclassified*  *Hyphomicrobiales* | 0 | 0 | 1577 | 1560 | 209 | 0 | 0 | 0 | 0 | 0 | 0 | 0 | 0 |
| *unclassified*  *Blastocatellia* | 0 | 0 | 0 | 0 | 0 | 0 | 0 | 0 | 0 | 0 | 0 | 0 | 0 |
| *unclassified*  *Bryobacterales* | 0 | 0 | 0 | 0 | 0 | 0 | 0 | 0 | 0 | 0 | 0 | 0 | 0 |

**REFERENCES**

1. Yang WH, McDowell AC, Brooks PD, Silver WL. New high precision approach for measuring 15N-N_2_ gas fluxes from terrestrial ecosystems. Soil Biol. Biochem. 2014; 69:234-241.

2. Xi D, Bai R, Zhang L, Fang Y. Contribution of anammox to nitrogen removal in two temperate forest soils. Appl. Environ. Microbiol. 2016; 82(15):4602-4612.

Fig. S1

Fig. S2

Fig. S3

Fig. S4

Fig. S5

Table S1

Table S2

Table S3

Table S4
